# Heterotopic pancreas in excluded stomach diagnosed after gastric bypass surgery

**DOI:** 10.1186/1471-2482-13-56

**Published:** 2013-11-23

**Authors:** Marta Guimarães, Pedro Rodrigues, Gil Gonçalves, Mário Nora, Mariana P Monteiro

**Affiliations:** 1 Department of General Surgery, Hospital de São Sebastião, Santa Maria da Feira, Portugal; 2 Endocrinology Unit of Hospital São Sebastião, Hospital de São Sebastião, Santa Maria da Feira, Portugal; 3 Department of Anatomy, Multidisciplinary Unit for Biomedical Research (UMIB), ICBAS, University of Porto, Porto, Portugal

**Keywords:** Heterotopic pancreas, Gastric bypass, Excluded stomach, Incretins

## Abstract

**Background:**

Heterotopic pancreas is defined as finding of pancreatic tissue without anatomic and vascular continuity with the normal pancreas. Heterotopic pancreas is a rare condition difficult to diagnose and with controversial clinical management.

**Case presentation:**

We describe a 43 year old female patient previously submitted to laparoscopic gastric bypass for primary treatment of morbid obesity; 5 years later, the patient was discovered to have a mass in the antrum of the excluded stomach that was found to be heterotopic pancreatic tissue. Before gastric bypass surgery, the presence of the pancreatic mass in the gastric wall was unnoticed in the imagiologic records.

**Conclusion:**

This is the first reported case of pancreatic heterotopy diagnosed in the excluded stomach after gastric bypass. A putative role of incretin hormones in mediating pancreatic cell hyperplasia of heterotopic pancreatic remnants should be considered an additional hypothesis that requires further research.

## Background

Heterotopic pancreas was first described in 1727 by Schultz and is defined as the presence of pancreatic tissue whithout anatomic and vascular continuity with the normal pancreas [1]. It is found in 0.55-15% of autopsy specimens [2] being more common at the age of 30–50 years with a male predominance [3]. The heterotopic tissue can be found anywhere from the distal end of the esophagus to the colon, but mostly occurs in upper gastrointestinal tract: stomach(25%), duodenum(30%) and jejunum(15%), but also in liver, gallbladder, distal small intestine, colon, appendix, omentum, fallopian tube, common bile duct, cystic duct, ampulla of Vater, spleen, lymph nodes and Meckel’s diverticula. Extra-abdominal sites included mediastinal cysts, bronchi, lung, umbilicus and brain [4-8]. The most common heterotopic site is the stomach (25-40%), especially antrum and prepyloric region on the greater curvature or posterior wall [9].

Heterotopic pancreas is thought to take place when the foregut rotates between weeks 5 and 8 of gestation, while pancreatic fragments detach from the pancreas and are deposited ectopically [10]. Heterotopic pancreatic tissue in the gastro-intestinal tract generally occurs as discrete firm, irregular, yellow nodules located in the submucosa. Histologically heterotopic pancreatic tissue is not a true neoplasm but rather a hamartoma of fat glandular tissue with pancreatic acinar formation and duct development [8]. In the majority of cases the patients are asymptomatic and the condition is incidentally discovered [11]. Almost all changes which can occur in the pancreas itself may develop in heterotopic pancreas [12], although malignant transformation is extremely rare [10].

There are several cases of heterotopic pancreases described in the literature, but to date none of them was diagnosed in the excluded stomach after gastric bypass.

## Case presentation

A 43 years old female patient was previously submitted to laparoscopic gastric bypass for morbid obesity without co-morbidities. Seven months after the bariatric surgery the patient showed intense episodic epigastric abdominal pain that was aggravated by food ingestion and led to multiple visits to the emergency room.

**Figure 1:**
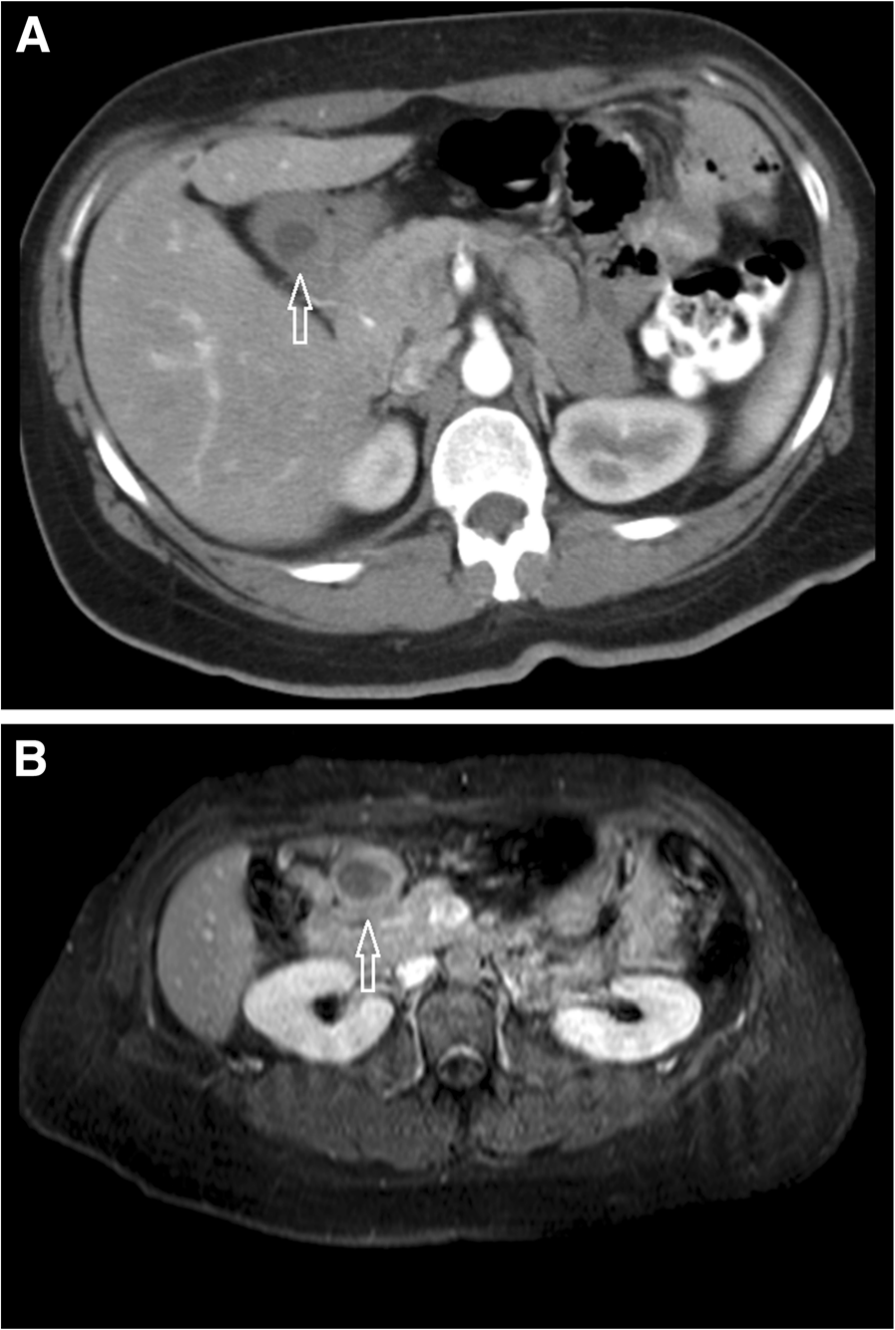
Abdominal MRI shortly after (A) and 5 years after gastric bypass (B) displaying the subserosal mass in the gastric antrum (arrows), the former previously unnoticed.

One month after the onset of pain complaints, the patient was offered hospital admission for further evaluation. Physical examination was unremarkable and routine blood assessment of liver and pancreatic functions were normal. The upper endoscopy and esophageal-gastro-jejunal transit were normal; the abdominal CT and MRI were considered normal despite the presence of a mass in the excluded stomach, as it was ascribed to the anatomical rearrangement after the bypass surgery (Figure 1A). During hospital stay the patient did present any evidence of abdominal pain, complaints or need for analgesia; after formal psychiatric evaluation, a major depression was diagnosed and the patient was started on anti-depressants.

Five years after gastric bypass, due to ongoing epigastric pain complaints, abdominal CT and MRI were repeated, with subsequent diagnosis of a 4.5 cm of greater diameter subserosal neoplasm in the antrum (Figure 1B).

**Figure 2:**
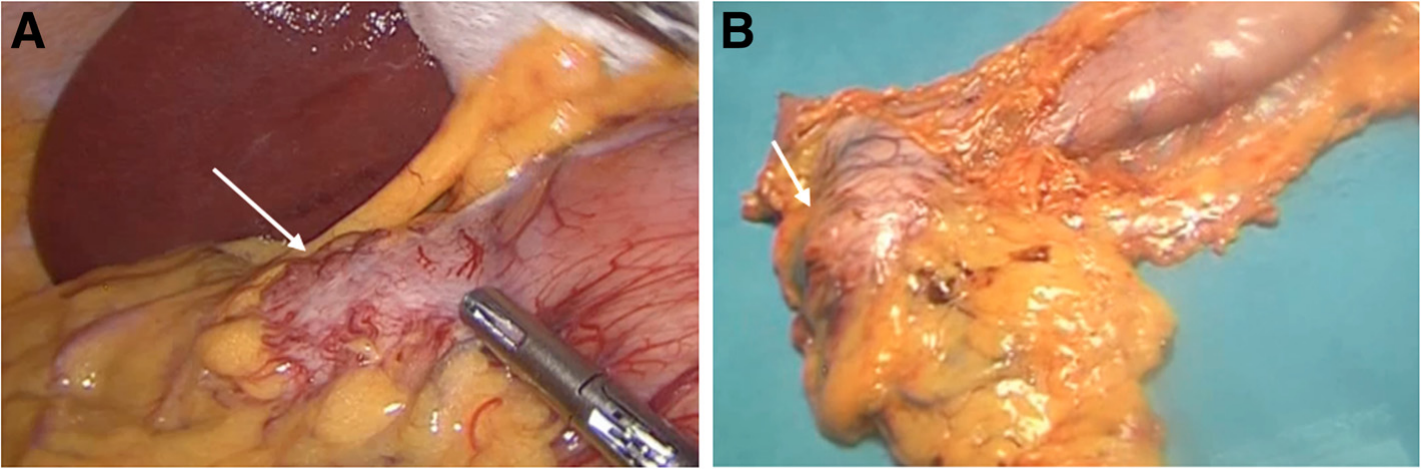
Macroscopic appearance of the subserosal mass at laparoscopy (A) and in the excluded stomach removed after partial gastrectomy (white arrows) (B).

**Figure 3:**
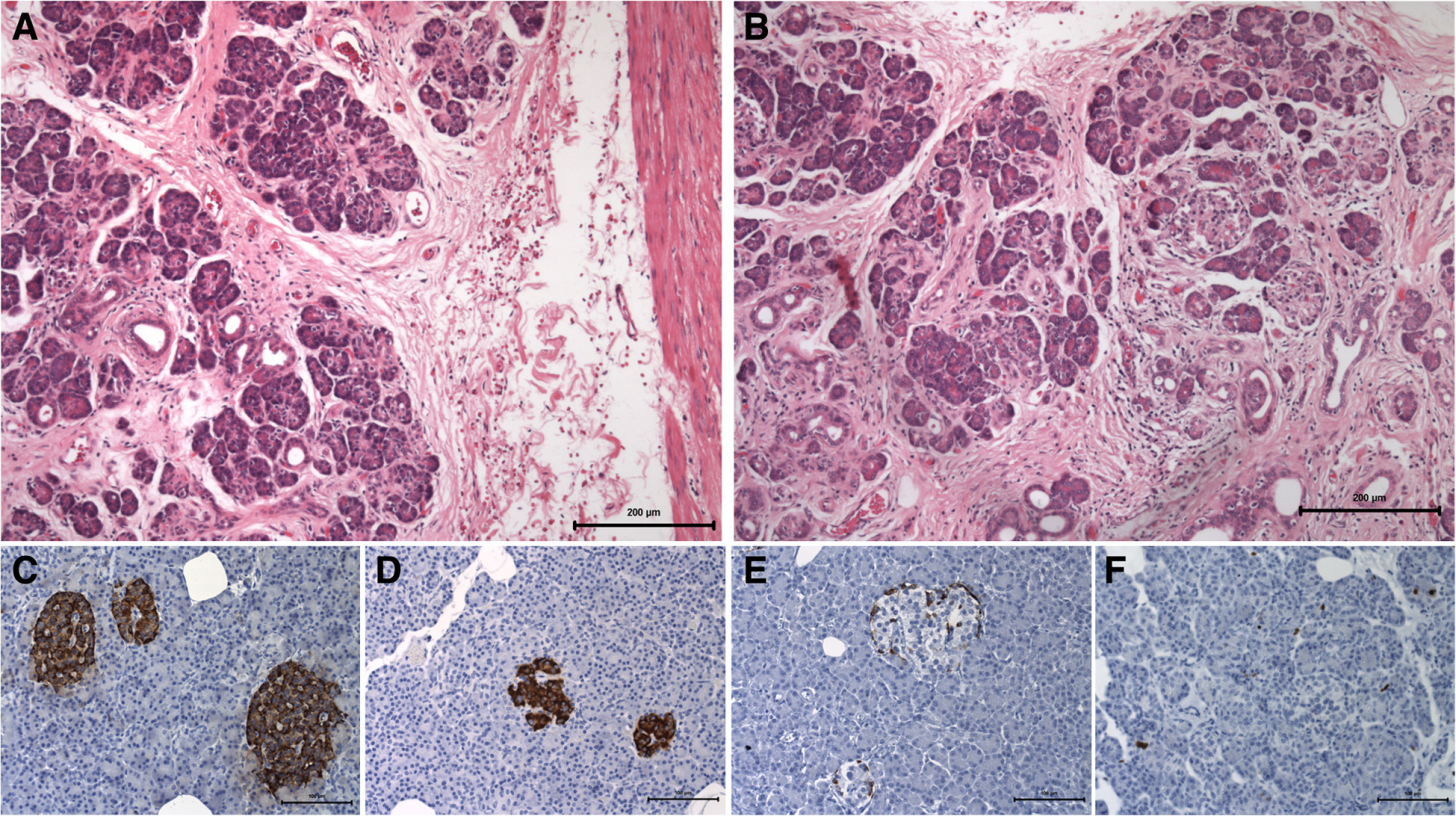
Histology of the muscular gastric wall and the normal heterotopic pancreatic tissue in the subserosa (A) and tissue nodule in the omentum (B), with respective imunostaining for the neuro-endocrine markers chromogranin A (C), insulin (D) and glucagon (E), showing normal exocrine pancreas and Langerhans islets, with normal distribution of neuroendocrine cells, as well as insulin and glucagon expressing cells; the proliferation index was also normally low as displayed by the Ki-67 staining (F) (immune staining in brown).

The patient underwent laparoscopic gastrectomy of the excluded stomach for suspected gastrointestinal stromal tumor (GIST) (Figure 2A). Gross examination of the specimen revealed a subserosal polypoid mass in the gastric antrum, which corresponded to a 4.5 cm cystic cavity of greater diameter with creamy yellowish thick content, growing in dependency of the gastric muscular layer (Figure 2B). The histology of the mass showed a flap of gastric wall with antral mucosa and a heterotopic pancreatic cist, while in the adipose tissue of the root of the greater omentum six other yellow and lobulated nodules were identified and dissected. All fragments corresponded histologicaly to pancreatic tissue with normal exocrine and endocrine distribution, as displayed by the immunohistochemistry staining for chromogranin A, insulin and glucagon expressing cells, as well as a low proliferation index as revealed by the Ki-67 staining, which are characteristic of the normal pancreatic tissue (Figure 3, A-F). After gastrectomy, the patient became asymptomatic and so has remained ever since.

## Discussion

Most patients with heterotopic pancreas are asymptomatic; when present, the reported symptoms include abdominal pain, epigastric discomfort, bleeding, nausea and vomiting; sometimes symptoms are related to complications, such as mechanical occlusion - intussusceptions and obstruction of the small bowel, obstructive jaundice and pyloric stenosis [8,13]. Other complications include: pancreatitis, pseudocyst formation, carcinomas, islet-cell tumors and inflammatory pseudotumors [6]. Most cases of gastric heterotopic pancreas are incidental findings during surgery, gastrointestinal exam or autopsy.

The differential diagnosis includes GIST, gastrointestinal autonomic nerve tumor (GANT), carcinoid, lymphoma or gastric carcinoma which can be misinterpreted on imaging studies or endoscopic examinations [14,15].

Since heterotopic pancreas is an uncommon condition, unlike GIST, it is rarely considered a diagnostic hypothesis. Five CT features were pointed as significant predictors in the differential diagnosis of heterotopic pancreas from GIST and leiomyomas, namely: 1) prominent enhancement of the overlying mucosa, 2) location, 3) long diameter/short diameter ratio of the lesion, 4) growth pattern and 5) lesion border [16]. At endoscopy the heterotopic pancreas generally appears as a well-defined dome-shaped filling defect with central umbilication. Definitive diagnosis requires histological confirmation.

Management of this condition is controversial. Most cases undergo surgery due to diagnostic uncertainty. Reasons for surgical treatment depend on the presence of symptoms, as well as need of definitive diagnosis, excluding malignancy or avoiding complications [17].

This patient had both normal abdominal ultrasound and gastroduodenoscopy before gastric bypass¸ and the presence of a mass in the excluded stomach had been unnoticed in the abdominal CT and MRI performed four years before gastrectomy. In the herein case presented, GIST of the excluded stomach was the first diagnostic hypothesis, due to the previous history of gastric bypass, endoscopic ultra-sound (EUS) could not be useful.

There was no evidence of acute pancreatitis in any recurrency of episodic abdominal pain that lead patient to visit the emergency room, as suggested from serum amylase and lipase levels, however, since the heterotopic pancreas included a cystic cavity with a thick liquid content, the formation of a cyst in result of retention of exocrine secretions in the absence of communication between the glandular epithelium and the gastric lumen cannot be excluded.

Gastric bypass is known to induce changes in the secretion of insulinotropic enteric hormones, which may be involved in metabolic changes, remission of diabetes mellitus [18,19] and have a role in inducing pancreatic exocrine and endocrine cell proliferation [20]. Thus, it is conceivable that microscopic heterotopic nodules of pancreatic tissue can grow in result of gastric bypass surgery, similarly to what has been hypothesized to occur with regards to orthotic pancreatic tissue. Notwithstanding, the action of incretin hormones in stimulation the growth of pancreatic tissue still needs further research.

## Conclusion

Heterotopic pancreas is a rare condition, difficult to diagnose and with controversial management. We present the first case of pancreatic heterotopy diagnosed after gastric bypass surgery. The role of incretin hormones in stimulating the growth of pancreatic cells and its consequences justifies further investigation.

## Consent

Written informed consent was obtained from the patient for publication of this Case report and any accompanying images. A copy of the written consent is available for review by the Editor of this journal.

## Abbreviations

CT: Computed tomography; EUS: Endoscopic ultra-sound; GANT: Gastrointestinal autonomic nerve tumor; GIST: Gastrointestinal stromal tumor; MRI: Magnetic resonance Imaging.

## Competing interests

The authors declare that they have no competing interests.

## Authors’ contributions

MG, PR, MN, GG have been made substantial contributions to diagnosis and treatment of the patient; MPM contributed with patient data analysis and interpretation; MG and MPM wrote the manuscript. All authors read and approved the final manuscript.

## Pre-publication history

The pre-publication history for this paper can be accessed here:

http://www.biomedcentral.com/1471-2482/13/56/prepub
